# Detection of Q Fever Specific Antibodies Using Recombinant Antigen in ELISA with Peroxidase Based Signal Amplification

**DOI:** 10.1155/2014/707463

**Published:** 2014-03-12

**Authors:** Hua-Wei Chen, Zhiwen Zhang, Erin Glennon, Wei-Mei Ching

**Affiliations:** Viral and Rickettsial Diseases Department, Naval Medical Research Center, Silver Spring, MD 20910, USA

## Abstract

Currently, the accepted method for Q fever serodiagnosis is indirect immunofluorescent antibody assay (IFA) using the whole cell antigen. In this study, we prepared the recombinant antigen of the 27-kDa outer membrane protein (Com1) which has been shown to be recognized by Q fever patient sera. The performance of recombinant Com1 was evaluated in ELISA by IFA confirmed serum samples. Due to the low titers of IgG and IgM in Q fever patients, the standard ELISA signals were further amplified by using biotinylated anti-human IgG or IgM plus streptavidin-HRP polymer. The modified ELISA can detect 88% (29 out of 33) of Q fever patient sera collected from Marines deployed to Iraq. Less than 5% (5 out of 156) of the sera from patients with other febrile diseases reacted with the Com1. These results suggest that the modified ELISA using Com1 may have the potential to improve the detection of Q fever specific antibodies.

## 1. Introduction

Q fever is a worldwide zoonotic disease caused by infection with* Coxiella burnetii*. This agent is highly infectious for humans by aerosol, where a single organism can cause the disease. Due to Q fever's worldwide distribution and the high infectivity of* C. burnetii*, US military and civilian personnel deployed overseas are at high risk of being infected. Several studies [1–3] showed that Q fever poses a greater threat to US forces deployed in Iraq than previously predicted. An investigation of febrile illness outbreak among marines in Hit, Iraq, highlights the fact that Q fever is capable of causing localized outbreaks in exposed military personnel with attack rates up to 50% and perhaps higher [4]. The U.S. Army Center for Health Promotion and Preventive Medicine (USACHPPM) initiated a Q fever surveillance program in early 2007. Over 150 cases have been confirmed among U.S. military personnel deployed to Iraq since 2007 [5]. In addition, the largest known reported Q fever outbreak involved approximately 4,000 human cases and occurred from 2007 to 2010 in the Netherlands [6].

Acute Q fever illness most commonly presents as a flu-like illness, pneumonia, or hepatitis. Symptoms of Q fever are easily confused with those due to a variety of other pathogens (e.g., dengue, malaria, and leptospirosis) that may require different treatment regimens. The chronic form is infrequent (<5% of patients with acute infections), but the potentially consequent endocarditis is often fatal if left untreated [6, 7]. Therefore, early diagnosis to guide an appropriate treatment is critical for patient care.

Although the presence of* C burnetii* DNA can be detected occasionally in patient serum of acute phase Q fever with Polymerase Chain Reaction (PCR) [8–10], the current diagnosis of Q fever relies mainly on serological methods [11]. These methods include the indirect immunofluorescent antibody assay (IFA) [12, 13], the complement fixation assay (CFA) [14, 15], and the enzyme-linked immunosorbent assay (ELISA) [16, 17]. Among these tests, IFA is considered to be the reference test during an endemic situation [8, 18]. Because the screening of serum samples by IFA was laborious, ELISA was used during an epidemic situation because it can be automated and is easier to perform [19]. The antigen used in ELISA is mostly whole cell preparation of phase I or phase II* C. burnetii* [16, 17, 19, 20]. Due to the hazard and difficulty of culturing and purifying* C. burnetii* in a biosafety level (BSL)-3 laboratory, the antigens are not available in most clinical laboratories. Although there are commercially available IFA and ELISA tests for Q fever, the serological test results vary considerably among different laboratories using the same kit. This may be due to the residual egg yolk or tissue culture proteins in the whole cell antigen preparation [2, 21]. Earlier studies focused on the identification of immunogenic antigens of* C. burnetii* discovered protein immunogens of molecular weights from 13 to 92 kDa [22, 23]. Among them, Hsp60, Com1, Cbmip, P1, and AdaA have been cloned and characterized previously [23–27]. More recently, several proteomic studies have identified additional immunogenic proteins by 2D-gel immunoblotting [28, 29] and protein microarray approaches [30, 31]. A 27 kDa immunodominant antigen Com1 was identified by all different methods mentioned above. In this study, we cloned and purified the Com1 antigen. 33 Q fever patient sera, 10 normal human sera, and 156 other febrile patient sera were used to evaluate the usage of recombinant Com1 antigen for the detection of Q fever specific antibodies in ELISA. The results demonstrated the amplification of ELISA signal may have the potential to improve the serological assay.

## 2. Materials and Methods

### 2.1. Bacterial Strains and Vectors

*Escherichia coli* Top10 (Invitrogen, CA) was used for general cloning. The cloned genes were inserted into pET24a (Novagen, CA) for the expression of Com1 protein.* E. coli* BL21 (DE3) (Invitrogen, CA) was used for expression of proteins under the control of phage T7* lac* promoter [32].

### 2.2. Cloning of the Gene Coding for the Com1 Protein into the Expression Vector pET24a

A primer pair [com1f (5′-CGGGATCCGCCCCCTCTCAATTCAGTTTTT-3′) and com1r (5′-AAGAATGCGGCCGCCTTTTCTACCCGGTCGATTTCT-3′)] was designed by using the nucleotide sequence of the open reading frame for the Com1 from strain RSA 493 (GenBank accession number NC002971.1). The coding sequence for amino acids 22 to 252 of the Com1 protein was amplified by PCR using genomic DNA isolated from* C. burnetti* RSA 493 strain as the template. The PCR product was digested with BamHI and NotI and ligated into the expression vector pET24a. Top10 competent cells were transformed with the ligation mixture, and colonies were screened for the presence of inserts with the right size. The final sequences were confirmed by DNA sequencing of the resulting plasmid.

### 2.3. Expression and Purification of the Recombinant Com1 Protein (rCom1)

*E. coli* BL21 (DE3) was transformed with plasmids carrying the Com1 insert. The recombinant* E. coli* colony with high expression level of the Com1 protein was grown overnight in Overnight Express medium TB (EMD Biosciences, CA) in the presence of kanamycin at 37°C with shaking at 200 rpm. Cell pellets from 500 ml cultures were resuspended in 20 ml of buffer A (20 mM Tris-HCl, pH 8.0, 1 mM EDTA, and 1 mM DTT) containing 2% deoxycholic acid (DOC). Cells extracted from sonication (Ultrasonic Liquid Processor model VirSonic 475, VIRTIS Company, NY) were centrifuged at 10,000 ×g for 30 min in a Thermo centrifuge (model IEC MultiRF). The pellets were resuspended in Hisbind buffer (20 mM Tris, pH 8.0, 0.5 M NaCl, 10 mM imidazole) containing 8 M urea by vortexing, placed on a shaker at room temperature for an additional 10 min, and centrifuged for 30 min at 10,000 ×g. The supernatant from one liter of culture was applied onto a 1 ml nickel-column (Ni-NTA) equilibrated with the same buffer containing 8 M urea. The recombinant protein was eluted from the column in a step gradient of 25, 50, 100, 200, 400, and 600 mM imidazole in Hisbind buffer containing 8 M urea. The eluates from 100, 200, and 400 mM imidazole were pooled. Refolding of rCom1 protein in 8M urea in Hisbind buffer was achieved by sequential dialysis as described previously [33].

### 2.4. ELISA with rCom1 Protein or Whole Cell Antigen

Different amounts (0.15, 0.3, 0.45, and 0.6 *μ*g per well) of rCom1 were used to coat the ELISA plate to determine the optimum amount for coating. The optimal amount of rCom1 was determined to be 0.3 *μ*g per well as coating plate with 0.45 and 0.6 did not increase the signal. The optimization was also performed for whole cell antigens and determined to be 0.15 *μ*g per well. The ELISA was performed and optical densities were measured as described previously [33].

### 2.5. Amplification of ELISA Signal by Streptavidin-Peroxidase Polymer

After incubation with diluted patient sera, the biotinylated anti-human IgM (Thermo Scientific, IL) or anti-human IgG (Santa Cruz Biotechnology, CA) at 1 : 5000 dilution in phosphate buffered saline (PBS) with 5% bovine serum albumin (BSA) was added. After 1 h of incubation at room temperature, the plates were washed as previously described then incubated with streptavidin-peroxidase polymer (Sigma, MS) at 1 : 8000 dilution in PBS with 5% BSA for another hour. At the end of incubation, the plates were washed again before the addition of the ABTS substrate. Optical densities at 405 nm (OD_405_) were measured after 30 minutes as previously described.

### 2.6. Human Sera

A panel of six positive sera with IFA titers was obtained from Panbio. 33 archived Q fever IFA positive patient sera were received from Naval Medical Research Unit number 3, Cairo, Egypt [4]. 10 archived normal human sera and 156 archived sera from patients with other febrile illness identified by their respective IFA (27 with scrub typhus, 45 with murine typhus, 56 with spotted fever-type rickettsioses, and 28 with oroya fever) were used as control (Tables 1 and 4). The study protocol was approved by the Naval Medical Research Center Institutional Review Board (case number PJT23) in compliance with all applicable Federal regulations governing the protection of human subjects.

## 3. Results

### 3.1. Expression and Purification of the rCom1 Protein

**Figure 1:**
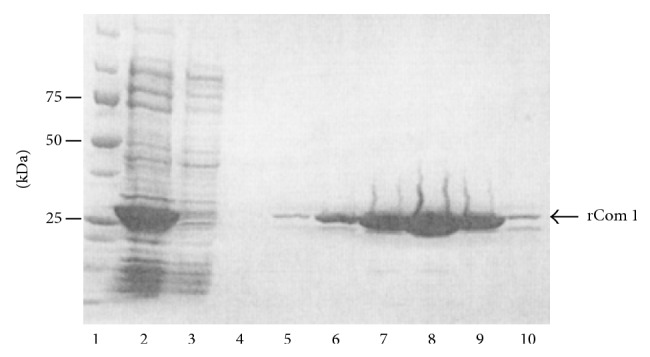
SDS-PAGE profile of the purification of rCom1 protein. rCom1 expressed in BL21 (DE3) and the fractions of the nickel-column were analyzed by electrophoresis. Lane 1, molecular weight marker; Lane 2, starting sample applied onto column; Lane 3, fraction of flow through; Lane 4, fraction of wash; Lane 5 to 10, fractions of the eluate with 25, 50, 100, 200, 400, and 600 mM imidazole.

The gene coding for amino acids 22 to 252 of Com1 protein was cloned into vector pET24a and was expressed as a histidine-Tag fusion proteins. The rCom1 was highly purified after a single step as a 27 kDa fusion protein (Figure 1).

### 3.2. Seroreactivity with rCom1 by ELISA

The refolded purified rCom1 was used as antigen to detect the presence of Q fever specific IgG and IgM in an ELISA. A panel of six positive serum samples (P1 to P6) with IFA titers and two normal serum samples (C1 and C2) was used. The results of ELISA showed only one sample (P4) had significant higher levels of specific IgG against rCom1 than the controls (Table 2) and none of them had detectable levels of specific IgM against rCom1 (data not shown). Sample P4 had IgG IFA titers equal or greater than 1024. It appears that by using rCom1 as the antigen, we can only detect the presence of specific IgG in samples with high IFA titer (greater than 1024).

### 3.3. Seroreactivity with rCom1 by Amplified ELISA

To improve the sensitivity of the ELISA, biotinylated anti-human IgG or IgM and streptavidin-peroxidase polymer were used. The amplified ELISA results for the same 8 samples are shown in Table 2. Of the six positive samples, six and four had greater signal levels of specific IgG and IgM against rCom1 than the controls, respectively. However, the IgM IFA titers and the amplified IgM ELISA signal values do not correlate very well.

### 3.4. Detection of Q Fever Specific Antibody in Sera of Patients Infected in Iraq

**Figure 2:**
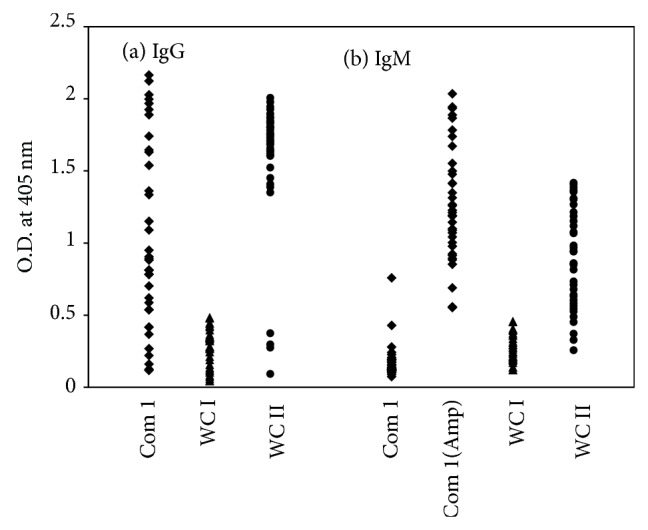
ELISA results for 33 US Marine sera with different antigens. IgG (a) and IgM (b) in patient sera specific for Com1 (square), whole cell phase I (triangle), and whole cell phase II (circle) were measured. (Amp): amplified ELISA. WC I: phase I whole cell antigen, WC II: phase II whole cell antigen. O.D., optical density.

33 Q fever serum samples confirmed by IFA were used to perform standard and/or amplified ELISA to detect specific antibody against rCom1 and standard ELISA to detect specific antibody against* C. burnetii* phase I and phase II whole cell antigen (Figure 2 and Table 3). Of those samples, 26 (79%) samples had specific IgG against phase I whole cell antigen, 33 (100%) samples had specific IgG against phase II whole cell antigen, and 29 (88%) had specific IgG against rCom1. The signals from IgG against rCom1 and phase II whole cell antigens in these samples were higher than those against phase I whole cell antigen (Figure 2(a)). For IgG measurement, Cohen's kappa values were 0.78 for rCom1 to phase II whole cell antigen and 0.67 for rCom1 to phase I whole cell antigen. In IgM ELISA, 26 (79%) samples had a specific antibody against phase I whole cell antigen, 31 (94%) samples had a specific antibody against phase II whole cell antigen, and 21 (64%) samples had a specific antibody against rCom1. With amplification, 29 samples had detectable specific antibody activity against rCom1 (Figure 2(b)). The kappa values were 0.65 for rCom1 (amplified) to phase II whole cell antigen and 0.55 for rCom1 (amplified) to phase I whole cell antigen in IgM assay. Less than 5% (5 out of 156) of other febrile illness samples had detectable IgG or IgM against rCom1 suggesting that rCom1 is Q fever specific (Table 4). These data indicate that the amplified ELISA with the rCom1 is specific and sensitive for the detection of antibodies against* C. burnetii*.

## 4. Discussion

The purified and refolded recombinant Com1 antigen without its signal peptide can be recognized by both human IgG and IgM to* C. burnetii* in ELISA. The recombinant protein antigen rCom1 offers a considerable advantage over the whole cell antigen of* C. burnetii*. It can be easily purified in large quantity and its quality can be more consistent from batch to batch. The amplified ELISA reported in this study involves the binding of biotinylated anti-human antibody (IgG or IgM) to the captured primary antibody followed by the addition of streptavidin labeled peroxidase polymers. The amplified method takes the advantage of the high affinity between streptavidin and biotin to form a stable complex and multiple peroxidases on each polymer chain to increase the peroxidase enzyme signal as compared to the standard ELISA. The additional step in the amplified ELISA does require longer processing time to perform the assay. Because skim milk contains endogenous biotin, it is incompatible with the amplification using streptavidin. BSA was used in the buffer for dilution of biotinylated anti-human antibodies and streptavidin-peroxidase polymer. This amplified method can also be used to determine IgG and IgM titers (data not shown).

By using the IFA as the reference assay, the rCom1 antigen can only detect the Q fever specific antibody in ELISA without amplification when the titer is greater than 1024, as indicated in Table 2. The amplification steps in ELISA yield a greater gap between the signal of the Q fever patient sera and the normal human sera (Table 2) or other febrile diseases patient sera (data not shown). With the amplification, we were able to detect Q fever specific antibody in some samples with very low IFA titers. In our study, close to 90% (29/33) Q fever patient sera had detectable IgG and IgM against rCom1. We were able to detect the Com1 specific IgG antibody in the majority (88%) of the Q fever patient sera without amplification, suggesting that those sera may have high IFA titers. Both IgG and IgM results of using rCom1 were in substantial agreement with that of phase II whole cell antigen as indicated by the kappa values, 0.78 and 0.65, respectively. There are fewer ELISA positive samples against phase I whole cell antigen than that of the phase II whole cell antigen. As demonstrated by previous studies, the phase I and phase II antigens interact differently with antisera collected in the early time of infection [34–37]. Phase II antigens can be recognized by both early (<20 days after infection) and late (>20 days after infection) infection-derived sera, while phase I antigens were recognized predominantly by antisera collected in the late time of infection. Since we do not have record of the sample collection date after onset of symptoms, we are unable to make the same conclusion. In our ELISA, all 33 Q fever sera were IgG positive but only 31 sera were IgM positive using whole bacteria as the antigens. It could be that those samples have very low IgM titers, as we observed in Table 2. 

The Com1 gene is highly conserved among the strains of* C. burnetii * [38]. The Com1 protein is a 27 kDa outer membrane-associated immunoreactive protein found in both acute and chronic disease strains of the pathogenic bacteria* C. burnetii*. It contains a CXXC motif that is homologous to the catalytic site of protein disulfide oxidoreductases [23]. Zhang et al. [39] reported the detection of IgG in human patient sera using a partially purified rCom1. In this study, we were able to produce rCom1 with greater than 95% purity to eliminate nonspecific signal due to impurity and detect both IgG and IgM with signal amplification in ELISA. The results showed that recombinant Com1 can detect Q fever specific antibodies. It is unrealistic to think that one antigen can detect all the antiserum. More well-characterized positive and negative samples from different parts of the world are needed to further evaluate the performance of rCom1. Additional antigens may be required to improve the assay's sensitivity.

## Figures and Tables

**Table 1:** List of samples used to perform ELISA with rCom1 and whole cell antigens.

Origin	Number	Diagnosis	Mode of original testing
Panbio	6	Q fever	Q fever IgG and IgM IFA (phase I and II)

NAMRU-3	33	Q fever	Q fever IgM IFA (>1 : 50) phase II Arboviral antibody testing negative for West Nile virus, Rift Valley fever, and sand fly fever (Naples and Sicilian types)

NMRC	10	Normal	None

**Table 2:** Comparison of the standard ELISA and amplified ELISA with rCom1 and IFA with whole cell antigens (WC I^b^ and WC II^b^) in normal human serum samples (C1 and C2) and patient samples (P1 to P6).

	ELISA (O.D.)^a^	Amplified ELISA (O.D.)^a^		IFA (titers)			
Sample	IgG	IgG	IgM	IgG		IgM	
	rCom1	rCom1	rCom1	WC I	WC II	WC I	WC II
C1	0.056	0.190	0.530	N/A	N/A	N/A	N/A
C2	0.074	0.203	0.536	N/A	N/A	N/A	N/A
P1	0.099	1.105	0.732	1024	512	40	40
P2	0.044	0.619	0.658	32	128	10	10
P3	0.048	0.611	1.434	128	128	10	10
P4	0.489	1.813	1.107	1024	2048	320	640
P5	0.069	0.808	0.699	512	512	40	20
P6	0.070	0.658	0.056	512	512	10	10

^a^The values represent the averages of two experiments.

^b^WC I: phase I whole cell antigen; WC II: phase II whole cell antigen.

**Table 3:** Detection of *Coxiella burnetii* specific antibodies in ELISA using different antigens in 33 Q fever patients.

Antigen	IgG positive^a^	(%)	IgM positive^a^	(%)
WC I^b^	26	79	26	79
WC II^b^	33	100	31	94
rCom1	29	88	21 (29)^c^	64 (88)^c^

^a^O.D. values higher than the cutoff values are considered as positive. The IgG cutoff values (means of 10 negative controls plus 2.3 standard deviations) are 0.168, 0.345, and 0.219 for WCI, WCII, and rCom1. The IgM cutoff values (means of 10 negative controls plus 2.3 standard deviations) are 0.190, 0.371, 0.121, and 0.689 for WCI, WCII, rCom1, and rCom1 (amplified).

^b^WC I: phase I whole cell antigen; WC II: phase II whole cell antigen.

^c^Based on the amplified ELISA results.

**Table 4:** List of archived samples used to determine the specificity of the rCom1 ELISA.

Origin	Disease	Number	No^a^ (%) positive
France	Murine typhus	20	1 (5)
France	Spotted fever-type rickettsioses	26	1 (4)
Peru	Oroya fever	28	0 (0)
Taiwan	Scrub typhus	27	1 (4)
Thailand	Murine typhus	25	1 (4)
Thailand	Spotted fever-type rickettsioses	30	1 (3)

^a^Sera that have antibody (IgG or IgM) levels above cutoff in amplified method.
